# Current and Emerging Potential of Magnetoencephalography in the Detection and Localization of High-Frequency Oscillations in Epilepsy

**DOI:** 10.3389/fneur.2017.00014

**Published:** 2017-01-30

**Authors:** Eleonora Tamilia, Joseph R. Madsen, Patricia Ellen Grant, Phillip L. Pearl, Christos Papadelis

**Affiliations:** ^1^Fetal-Neonatal Neuroimaging and Developmental Science Center, Division of Newborn Medicine, Department of Medicine, Boston Children’s Hospital, Harvard Medical School, Boston, MA, USA; ^2^Division of Epilepsy Surgery, Department of Neurosurgery, Boston Children’s Hospital, Harvard Medical School, Boston, MA, USA; ^3^Division of Epilepsy and Clinical Neurophysiology, Department of Neurology, Boston Children’s Hospital, Harvard Medical School, Boston, MA, USA

**Keywords:** high-frequency oscillations, magnetoencephalography, epilepsy, epileptogenic zone, epilepsy surgery, source localization

## Abstract

Up to one-third of patients with epilepsy are medically intractable and need resective surgery. To be successful, epilepsy surgery requires a comprehensive preoperative evaluation to define the epileptogenic zone (EZ), the brain area that should be resected to achieve seizure freedom. Due to lack of tools and methods that measure the EZ directly, this area is defined indirectly based on concordant data from a multitude of presurgical non-invasive tests and intracranial recordings. However, the results of these tests are often insufficiently concordant or inconclusive. Thus, the presurgical evaluation of surgical candidates is frequently challenging or unsuccessful. To improve the efficacy of the surgical treatment, there is an overriding need for reliable biomarkers that can delineate the EZ. High-frequency oscillations (HFOs) have emerged over the last decade as new potential biomarkers for the delineation of the EZ. Multiple studies have shown that HFOs are spatially associated with the EZ. Despite the encouraging findings, there are still significant challenges for the translation of HFOs as epileptogenic biomarkers to the clinical practice. One of the major barriers is the difficulty to detect and localize them with non-invasive techniques, such as magnetoencephalography (MEG) or scalp electroencephalography (EEG). Although most literature has studied HFOs using invasive recordings, recent studies have reported the detection and localization of HFOs using MEG or scalp EEG. MEG seems to be particularly advantageous compared to scalp EEG due to its inherent advantages of being less affected by skull conductivity and less susceptible to contamination from muscular activity. The detection and localization of HFOs with MEG would largely expand the clinical utility of these new promising biomarkers to an earlier stage in the diagnostic process and to a wider range of patients with epilepsy. Here, we conduct a thorough critical review of the recent MEG literature that investigates HFOs in patients with epilepsy, summarizing the different methodological approaches and the main findings. Our goal is to highlight the emerging potential of MEG in the non-invasive detection and localization of HFOs for the presurgical evaluation of patients with medically refractory epilepsy (MRE).

## Introduction

Epilepsy is one of the most common neurological disorders affecting children and adults (1, 2). About 65 million people currently carry the diagnosis of epilepsy all over the world (3), and in the US only, epilepsy causes about $9.6 billion of medical expenditures and indirect care (4). Although most of the patients are treated successfully with antiepileptic drugs, approximately 30% of patients suffer from medically refractory epilepsy (MRE), i.e., they have unsatisfactory control and continue having seizures (5, 6). For these patients, epilepsy surgery represents the most promising treatment to pursue seizure freedom.

**Figure 1:**
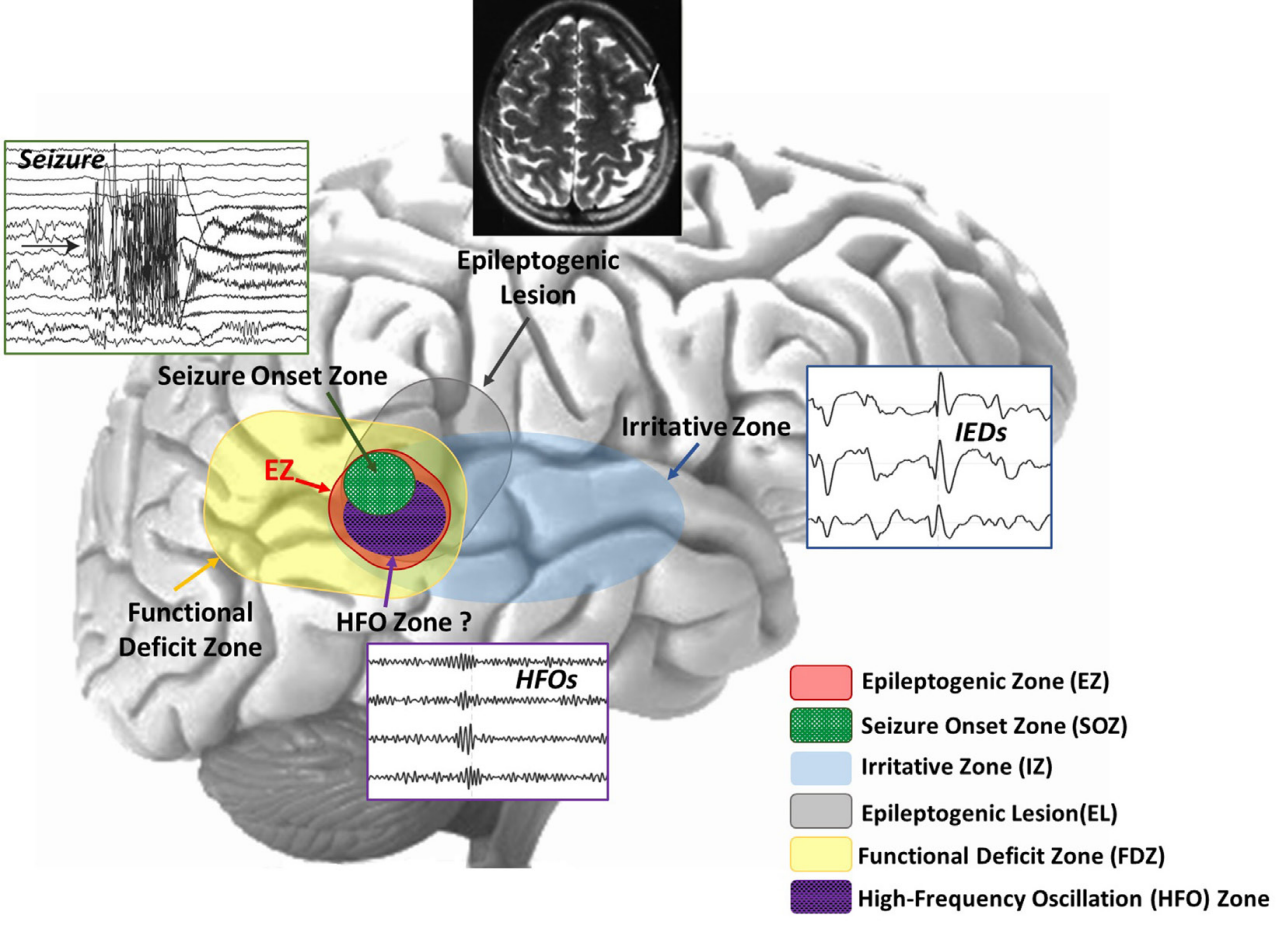
**Schematic representation of the overlapping cortical zones in epilepsy**. Different cortical zones are estimated by the epileptologists during the presurgical evaluation of a patient with epilepsy. These zones can often overlap, providing the epileptologist with concordant findings for the delineation of the epileptogenic zone (EZ). The high-frequency oscillation (HFO) zone is another potentially epileptogenic area that has been recently added to this picture as a further piece of information to circumscribe the EZ.

The objective of epilepsy surgery is the complete resection or disconnection of the epileptogenic zone (EZ), the area of the cortex necessary for the generation of habitual seizures and the smallest amount of tissue that can be removed to achieve a seizure-free outcome (7, 8). The only way to evaluate the success of the epilepsy surgery is by looking at the postsurgical outcome: if the EZ has been correctly identified and resected with no damage to the functionally relevant eloquent cortex (i.e., the region that is necessary for defined cortical functions), the patient will be seizure free with minimal or no functional deficits. Hence, the success of epilepsy surgery strongly depends on the successful delineation of the EZ. However, there is no diagnostic modality able to unambiguously delineate this zone (8). The EZ is a theoretical construct, and to date, there is no established marker that definitively determines its location and extent. The EZ can only be estimated through a variety of diagnostic tests that point out different cortical zones that are considered more or less precise indicators of the EZ (8–10) (see Figure 1):

– The *seizure-onset zone (SOZ)*, i.e., the area where the clinical seizures originate on ictal recordings.
– The *irritative zone*, i.e., the area of the cortex that generates interictal epileptiform discharges (IEDs) in the electroencephalography (EEG) or magnetoencephalography (MEG).
– The *epileptogenic lesion*, i.e., a structural brain abnormality that is causally related to the epilepsy.
– The *functional deficit zone*, i.e., the area of the cortex that is functionally abnormal during the interictal period, as indicated by neurological examination, neuropsychological testing and functional imaging or non-epileptiform EEG or MEG abnormalities.

The SOZ is generally considered the best estimate of the EZ, and it is removed in the majority of epilepsy surgeries. However, the identification of the SOZ is difficult because clinical seizures are unpredictable in nature and thus can be difficult to be captured by EEG. In addition, seizures captured during patient’s monitoring may not represent the full extent of the EZ. This is why the removal of the entire SOZ does not always lead to successful outcome. Another indicator of the EZ is the irritative zone, which is defined by the localization of IEDs that occur more frequently than seizures. The big advantage of the irritative zone is that it can be evaluated during the interictal period independently from the occurrence of seizures. This reduces the required recording time and the associated cost and patient’s discomfort. However, the irritative zone is often more widespread than the actual EZ and thus less specific to it (9). Furthermore, the presence of an epileptogenic lesion in the proximity of the irritative zone or the SOZ can provide an additional indicator of the location of the EZ.

Along with the localization of the EZ, the mapping of the eloquent cortex is essential to determine viability and strategy of the epilepsy surgery. This region is determined during the patient’s presurgical evaluation by identifying the essential functional areas that subserve motor, memory, language, and visual functions, which need to be preserved from resection. Thus, the ultimate goal of epilepsy surgery is to achieve seizure freedom by removing the EZ and also avoiding functional deficits caused by any damage to the eloquent cortex (10).

All the aforementioned cortical zones are initially defined using a battery of non-invasive diagnostic tests, such as scalp EEG, MEG, magnetic resonance imaging (MRI), function MRI (fMRI), positron emission tomography, and single photon emission computed tomography. The results of these non-invasive tests can be insufficiently concordant and fail to derive a clear hypothesis about the location of the EZ. In these cases, long-term monitoring with intracranial EEG (iEEG) is needed in order to evaluate the possibility of surgery (11–14). However, iEEG recordings face several limitations not only related to their invasiveness and cost but also to their limited spatial sampling that might lead to misleading conclusions (15, 16). The intracranial electrodes have a field of view of only several millimeters and, if their placement is not optimal, areas with relevant epileptic activity may be overlooked. Therefore, seizures that originate from areas not covered by the iEEG electrodes, but propagate to the area where the electrodes are placed, might lead to misleading results. In order to reduce or improve the use of such invasive, time-consuming, and costly investigations, and to improve the postsurgical outcome of patients who can undergo surgery, non-invasive presurgical tools with high sensitivity and specificity to the epileptogenic focus are being sought (17). Utilization of a non-invasive biomarker may help in various clinical scenarios that are typical in MRE (18), particularly in children. These include, for example, (i) patients with early onset severe epilepsies in which both generalized clinical semiology and EEG abnormalities can manifest yet with the presence of a definitive localized epileptogenic focus (19); (ii) tuberous sclerosis with multiple tubers in which resection of the most epileptogenic tuber can greatly improve seizure outcome (20); (iii) multifocal cortical dysplasia in which resection of the most epileptogenic lesion can lead to seizure freedom (21); (iv) bilateral migrational anomalies, e.g., polymicrogyria, in which partial lesionectomy may lead to seizure control and also enable sparing of eloquent cortex; and (v) temporal lobe epilepsy, whose large proportion of failed surgeries indirectly shows many underlying epileptogenic networks (18).

### New Promising Trends for the Delineation of the EZ

Given the lack of an unambiguous marker of the EZ and the limitations of the invasive recordings, the presurgical delineation of the EZ is complicated and often unsuccessful. As a result, a large proportion of patients undergoing epilepsy surgical resection [34–73% depending on the resection type (22)] continue to have seizures after the surgery. In addition, epilepsy surgery remains the most underutilized of all accepted medical interventions (23, 24): in United States, less than 1% of patients with MRE are referred to epilepsy centers for surgery (25). In order to improve the safety and efficacy of the epilepsy surgical treatment, there is an overriding need to identify and validate reliable biomarkers that can determine the extent and location of the EZ with high precision and accuracy.

During the last decade, high-frequency oscillations (HFOs) above 80 Hz have emerged as a new promising biomarker of epileptogenicity (26, 27). Recent studies have shown that the resection of the tissue generating HFOs may improve presurgical diagnosis (28, 29) and surgical outcome of patients with MRE (30–38). The HFO zone has been recently proposed as a further piece of information to circumscribe the EZ (see Figure 1). Its correlation with the other cortical zones is an area of active research (26, 27). Despite the promising findings, HFOs are not yet suited for the diagnosis or monitoring of epilepsy in clinical practice (39, 40). One of the major reasons is the difficulty to detect and localize them with non-invasive methods (26, 27, 40). However, as the clinical use of MEG has been increasing for patients with epilepsy (41–47), an emerging body of literature has provided evidence that HFOs can be also recorded non-invasively (48–52). These recent findings pave the way for the translation of these new biomarkers in clinical practice.

The use of a non-invasive biomarker of epileptogenicity might limit long-term monitoring and invasive intracranial recordings leading to a significant improvement of the presurgical evaluation procedure in MRE patients. Such a biomarker would not only help to identify the epileptogenic tissue for surgery but also permit definitive differential diagnosis of epilepsy from acute symptomatic seizures so treatment can begin immediately. It might also make it possible to assess the efficacy of therapeutic interventions without waiting for another seizure to occur, which could be associated with significant morbidity or mortality (40).

## MEG in the Presurgical Evaluation of Patients with Epilepsy

Magnetoencephalography is being increasingly used during the first phase of the presurgical evaluation of patients with MRE (41–47), helping to evaluate whether surgery is viable and to plan the surgical strategy. MEG is rapidly becoming invaluable for the presurgical evaluation of patients with epilepsy (17) due to its several strengths and possible applications:

- *Intrinsic properties*: MEG presents a unique set of intrinsic properties: (i) MEG signals are not distorted by the skull or the intervening soft tissues between brain and scalp (53–55); this property is particularly important in patients with large lesions, anatomical malformations, or patients who already had a resection and undergoing second surgery, (ii) MEG, unlike EEG, is contactless and thus patient’s preparation is faster; this is particularly important for pediatric patients who are difficult to stay still for long time and do not always follow instructions; (iii) MEG signals can be recorded with a high density of sensors avoiding the problem of salt bridge between EEG electrodes, a problem often faced particularly in children due to their small heads (41); and (iv) high-frequency activity is considered to be less susceptible to contamination from muscular activity in MEG than in scalp EEG (56, 57).
- *Functional mapping*: MEG has the ability to localize functional areas of the brain (58–63), such as primary sensory areas (i.e., visual, somatosensory, and auditory cortexes), as well as areas responsible for higher and more complex cognitive functions [i.e., language (64, 65) and memory (66, 67)]. MEG is advantageous compared to alternative methods, such as the intraoperative awake surgery (68) and the fMRI (69–71).
- *Optimization of iEEG*: MEG can guide the placement of iEEG electrodes (44, 72–74) by providing information regarding the localization of epileptic activity and functional areas. Such information regarding the electrodes placement is important since iEEG cannot be repeated easily due to the local scar following implantation. This may be particularly beneficial for patients with normal MRI findings (61, 75) or with multifocal/diffuse disease (45, 76).

In summary, MEG has several significant inherent strengths that make it a valuable tool to localize non-invasively the epileptic activity, to map the eloquent cortex, and to guide the placement of subdural strips and grids and/or depth electrodes. The advantages of MEG in the preoperative evaluation of patients with MRE have been reported in several studies that showed that the concordance of the MEG localization with the resected area correlated with postsurgical outcome (61, 77–79). The major limitation of MEG is the expense: MEG use is associated with high costs, first in terms of initial equipment purchase (~$1.5–2 million) and second in terms of daily operations (liquid helium and personnel expertise required) (41). The operational costs are expected to decrease significantly in the near future thanks to the availability of liquid helium recycling systems (41).

## Basic Principles of MEG

**Figure 2:**
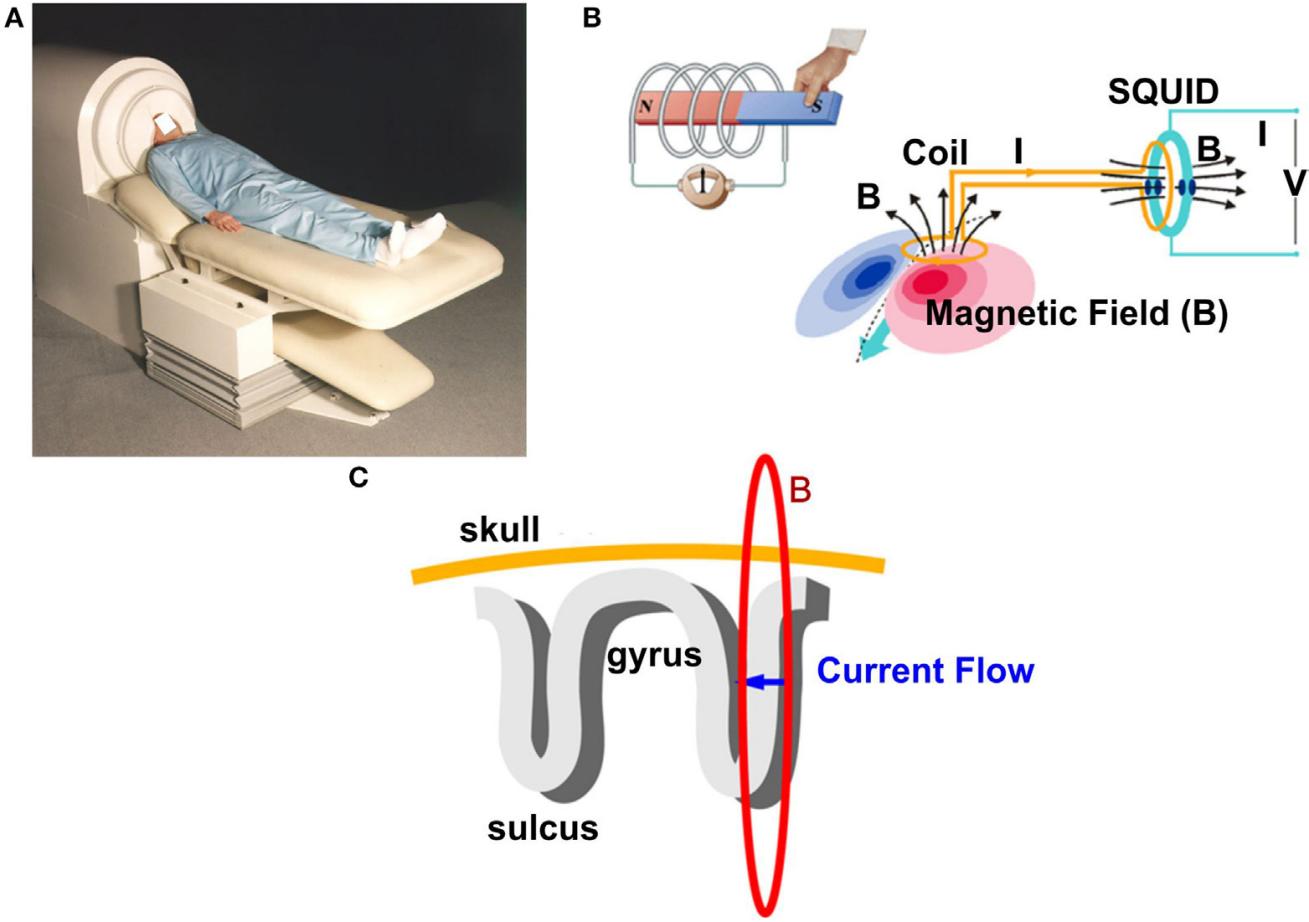
**Basic principles of signal generation in a conventional adult magnetoencephalography (MEG) system**. **(A)** A conventional MEG system. The patient places his/her head in a special helmet that accommodates a high number of sensors (typically >250). **(B)** Schematic representation of the electromagnetic induction phenomenon: a changing magnetic field (B) generates a measurable electrical current (I) that is recorded outside of the head by special magnetic field pick-up coils. The coils are connected with the superconducting quantum interference device (SQUIDs), which convert weak changes in the magnetic field into measurable voltage (V). **(C)** Schematic representation of the MEG signal generation. An intracellular electrical current (blue arrow) generates a magnetic field (red ring) around the apical dendrite. The magnetic field is picked up by the detection coils. Sources in the brain sulci cause tangential fields that can be detected by MEG.

Magnetoencephalography records the magnetic activity generated by electrical currents in active neurons of the human brain using the phenomenon of electromagnetic induction (41, 80, 81). Simultaneous recordings of epileptiform activity by MEG and iEEG have demonstrated that 4 cm^2^ of synchronously active cortex is needed to generate a spike that can be detected by MEG (82). The magnetic signals generated by the human brain are extremely weak compared to background electromagnetic activity. Thus, MEG recordings are performed inside specially designed rooms, called magnetically shielded rooms, which minimize the electromagnetic noise from external sources such as the power line or electrical devices. Figure 2 shows a conventional adult MEG system and its basic principles. During MEG recordings, the patient lays down on a bed and places his/her head inside the MEG sensor helmet. An illustrative complete description of MEG recordings can be found elsewhere (51). The MEG helmet contains specially designed detection coils that are able to record changes in the magnetic field (83, 84). These coils are connected to superconducting quantum interference devices, which convert the magnetic field passing through the detection coils into voltage changes.

Magnetoencephalography offers excellent temporal resolution in the range of sub-milliseconds and very good spatial resolution of few millimeters (85–89). Under specific requirements, MEG can localize superficial sources with an accuracy that reaches the cytoarchitectonic level of the cortex (90). Several studies reported that MEG and scalp EEG are comparable in terms of localization accuracy but their combination has better yield than either technique alone (89, 91). In epilepsy patients, some studies showed that MEG is more sensitive for spike sources, especially from the neocortex, because of the higher signal-to-ratio than EEG (89, 92), but no conclusions can be drawn yet. The relative orientation of the active source with respect to the skull significantly affects the strength of the recorded MEG signals. Sources with tangential orientation produce the maximum measurable magnetic field outside the scalp, while sources with radial orientation produce the minimum measurable magnetic field and thus are almost undetectable (93–95).

## Magnetic Source Imaging (MSI)

**Figure 3:**
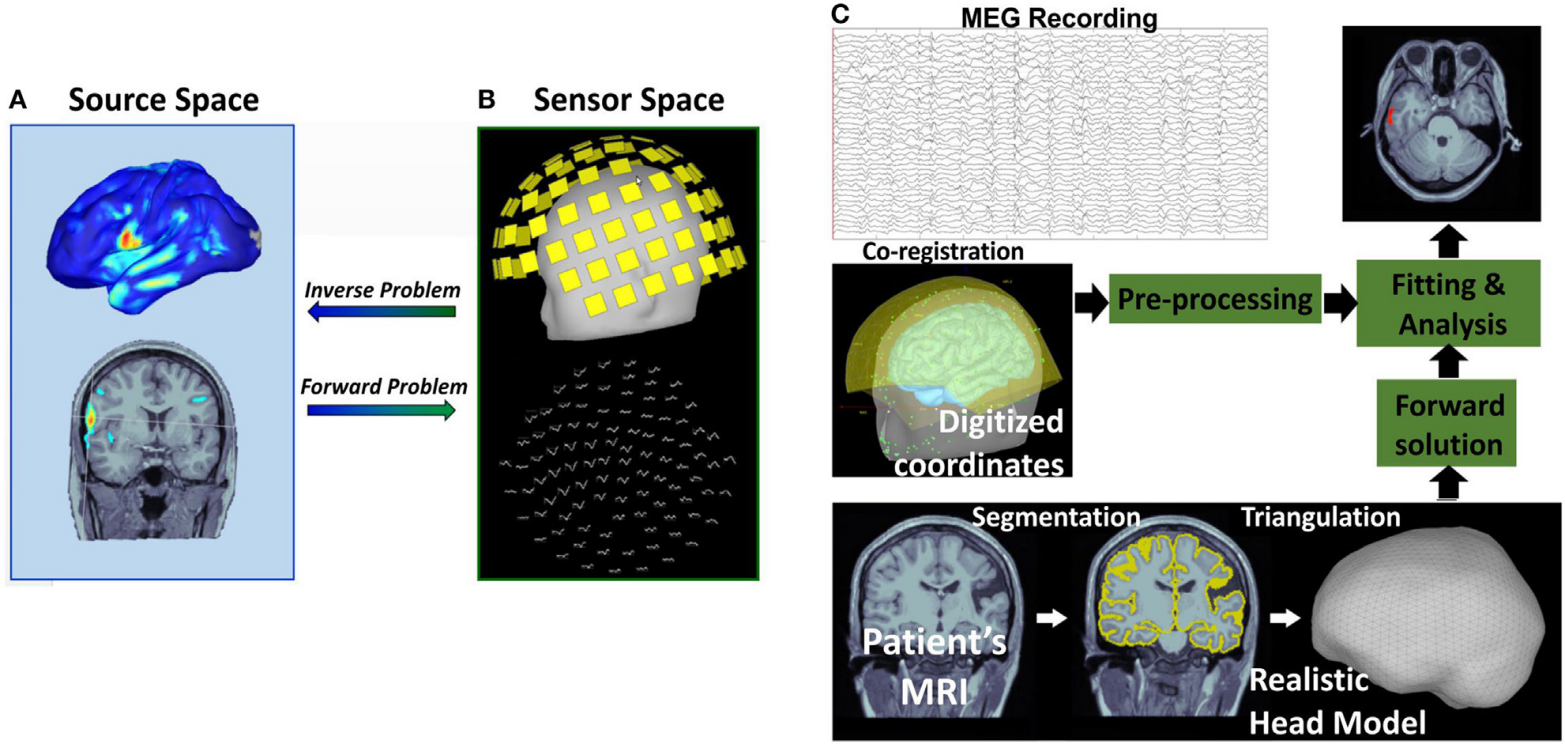
**Magnetic source imaging (MSI): inverse and forward problem**. The inverse problem consists in identifying the brain sources **(A)** that generate the observed magnetic field recorded by extracranial magnetoencephalography (MEG) sensors **(B)**. The forward problem explains how a known intracranial source **(A)** produces an extracranial distribution of magnetic activity **(B)** assuming a specific head model. Flow diagram of the MSI process is shown in **(C)**. MSI with a realistic head model requires three inputs: MEG recording, patient’s magnetic resonance imaging (MRI), and digitized coordinates of MEG electrodes.

Magnetoencephalography records the magnetic brain activity that is measurable outside the scalp using extracranial sensors. Thus, the number, location, and strength of the generators that produce the MEG signal are unknown. In order to define the underlying generators and estimate their characteristics, the inverse problem should be solved (81) (Figure 3). The inverse problem does not have unique solution because an infinite number of sources within the brain can produce similar extracranially recorded fields. Thus, *a priori* assumptions about the number and the nature of the underlying sources are necessary to constrain the inverse problem solution. Assumptions should also be made regarding the nature of the volume conductor, what is called the forward problem, by postulating *a priori* head models (96) (Figure 3). The forward problem has unique solution: an intracranial source of known location, orientation, and magnitude generates a mathematically well-defined extracranial magnetic field.

Magnetic source imaging is the method that combines MEG with MRI by projecting the magnetic activity recorded with the MEG on the patient’s anatomic image after solving the inverse problem (97). The MSI is a model-based imaging technique that integrates temporal and spatial components of MEG to identify the generating source of the magnetic fields recorded by the physical MEG sensors (see Figure 3). MSI significantly improves the interpretation of MEG raw data, which consist of the magnitudes of magnetic fields measured at different recording sites, since it enables to estimate the three-dimensional intracerebral location, orientation, and time activity of the underlying neuronal sources. MSI has both technical and clinical validity in the localization of the sources of IEDs (45, 97, 98).

## High-Frequency Oscillations as a new Biomarker of Epilepsy

High-frequency oscillations in epilepsy are generally described as short spontaneous EEG patterns in the frequency range from 80 to 500 Hz, consisting of at least four oscillations that stand out of the background activity (27). HFOs can be sub-classified according to their frequency content in “ripples” (80–250 Hz) and “fast ripples” (250–500 Hz) (99). Recently, very-high-frequency oscillations (>1,000 Hz) have been also observed in patients with epilepsy (100). Ripples and fast ripples seem to be generated by different pathophysiological mechanisms, but it is still debated whether their relation with the EZ is different. In literature, different recording methodologies (micro- vs. macro-electrodes) have been adopted (101), which led to discrepant conclusions (26) on the importance of discriminating between the two frequency ranges for the delineation of the EZ. Further research is needed to determine whether this discrimination is crucial or not. Furthermore, it may be challenging to reliably distinguish between physiological and pathological activity when looking at epileptic HFOs, since they may overlap with physiological oscillations in both ripple and fast ripple frequency bands (102, 103).

## HFOs in the Presurgical Evaluation of Epilepsy

High-frequency oscillations are considered a valuable piece of information that could enable a direct definition of the EZ (27, 104) (Figure 1). Recent and ongoing research has been investigating the correlation of the HFO generating area with respect to the other cortical zones [for reviews, see Ref. (26, 27, 105, 106)]. HFOs have proved to be a reliable interictal indicator of the SOZ: they have been observed in the SOZ with higher rates than outside during interictal periods (107–110). Also, HFOs seem to be more specific and accurate markers of the SOZ than interictal spikes (110). Interictal HFOs can overlap spikes but can also occur independently from them in space and time (109). The clinical significance of the distinction between HFOs with and without spikes has not been established yet. However, it has been suggested that considering only HFOs that overlap with spikes would be particularly beneficial in the clinical context because it allows (i) reducing the time needed for the inspection of EEG or MEG data and (ii) excluding physiological HFOs that would represent false detections of epileptogenic biomarkers (111). Furthermore, spikes with HFOs have shown to be more closely related to the SOZ than spikes in general (109). This has led to hypothesize that the presence of HFOs may be crucial to distinguish between “red” and “green” spikes, i.e., between pathological and non-pathological spikes originating, respectively, from inside or outside the EZ. The occurrence of HFOs may help to perform such distinction and identify the most epileptogenic spikes (112, 113). Further research is needed to investigate this HFO-spike concurrence in relation to epileptogenicity.

High-frequency oscillations can also help in the delineation of the EZ independently of lesional boundaries. HFOs have proved to be a more reliable marker of the SOZ than the epileptogenic lesion, since their occurrence within a lesion is more closely linked to the SOZ than to pathologic tissue changes (114). Furthermore, in patients with focal cortical dysplasia, HFOs have been found mainly in the SOZ rather than in other areas of pathologic tissue (115). Thus, they represent a marker of the SOZ independent of the underlying pathology.

Looking at the postsurgical outcome, HFOs have proved to be promising markers of epileptogenesis for a successful surgery: removal of the HFO generating tissue is an indicator of good surgical outcome (31–38), while residual HFOs after surgery can predict poor outcome (37). Furthermore, the surgical removal of all the HFO generating areas, both within and outside the SOZ, correlates with good postsurgical outcome, suggesting that HFOs can also point toward epileptogenic areas outside the SOZ (31).

In summary, HFOs seem to be a reliable interictal biomarker of tissue capable of generating seizures. However, the evidence for an effective clinical use of HFOs for epilepsy surgery decision making is still weak (116) and the application of HFOs for presurgical evaluation is at an early stage (117, 118). There are still significant challenges for the clinical translation of HFOs as epileptogenic biomarkers. One of these challenges is the difficulty to detect and localize them non-invasively. This is reflected in the current literature that mostly investigated HFOs using invasive intracranial recordings (26, 27).

### HFO Automatic Detection

To date, the visual detection of HFOs has led to a good understanding of the relationship between HFOs and epilepsy (26, 27, 108). However, visual marking is time consuming and subjective, since there is not a well-established definition of HFOs that enables their straightforward identification and each recorded signal should be inspected with an extended time scale display (i.e., 250 mm/s). The development of automatic HFO detectors has received much interest during the last decade and represents an area of active research (50, 51, 119–129). Such automatic detectors are crucial for the investigation of HFOs as biomarkers of epileptogenicity. They are likely necessary to propel future clinical applications, since they would enable an objective and consistent identification of HFOs in large-scale recordings.

**Figure 4:**
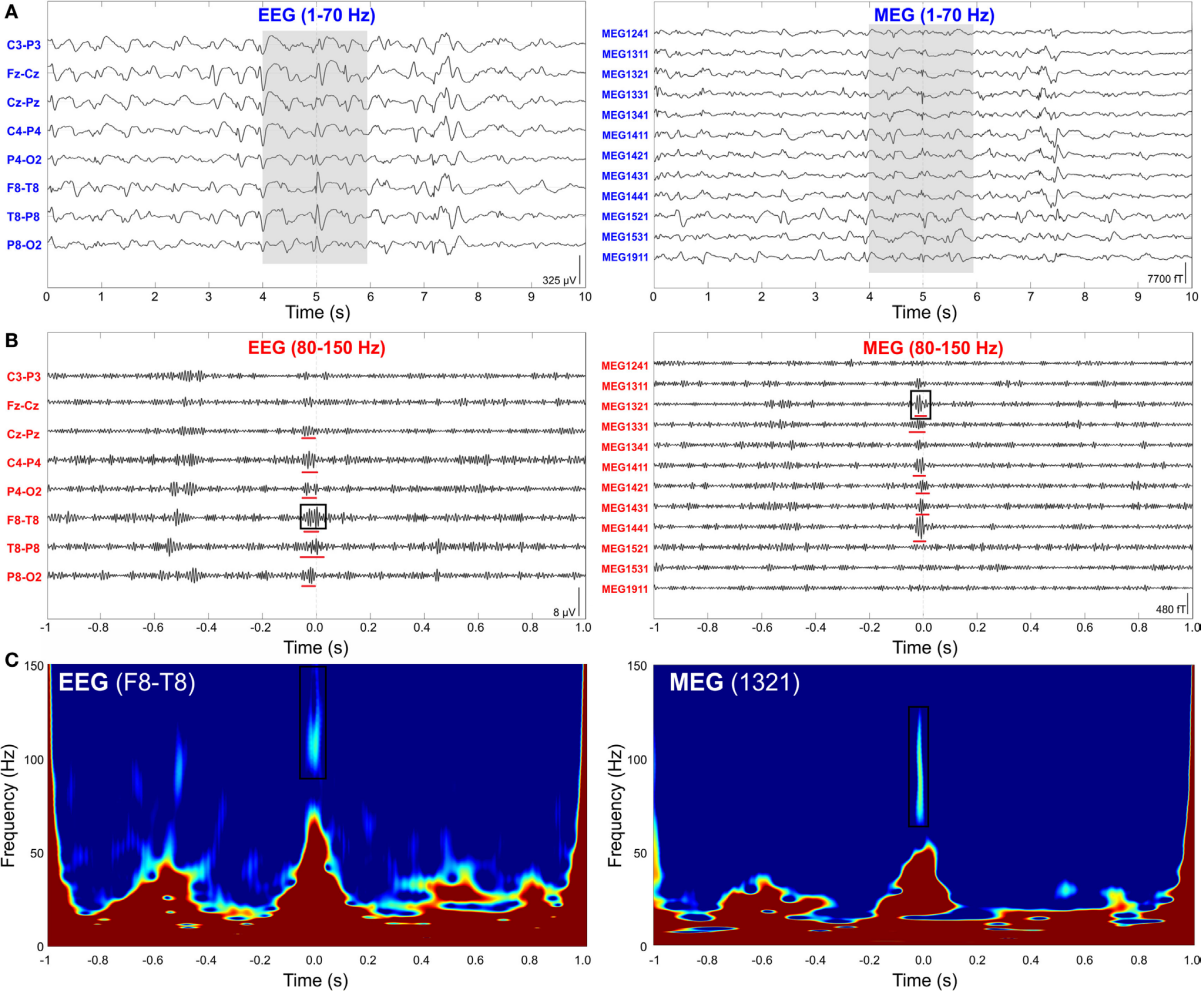
**High-frequency oscillations (HFOs) on simultaneous scalp electroencephalography (EEG) and magnetoencephalography (MEG) data**. **(A)** Interictal spikes from a pediatric patient (15-year-old girl with encephalomalacia of the right middle cerebral artery region) with medically refractory epilepsy simultaneously recorded with scalp EEG (left) and MEG (right). Ten seconds of data filtered from 1 to 70 Hz are shown. **(B)** Interictal HFOs co-occurring with spikes. Extended timescale display of 2 s of data highlighted with gray background in **(A)**. Data are filtered from 80 to 150 Hz for the HFO visualization. HFOs are underlined with red lines. **(C)** Time–frequency map of two representative HFOs marked with black squares in **(B)**. We can observe the isolated peak in the ripple frequency band. Source: adapted from Papadelis et al. (51).

Given the lack of a quantitative definition of HFOs, the criteria used for automated detection differ from study to study, as well as the reported range of HFO amplitude (10–1,000 µV) and duration (30–100 ms) (120). Several algorithms have been proposed in the last decade for the automatic detection of HFOs [for reviews, see Ref. (119, 120)]: they commonly define an HFO event as characterized by at least four oscillations standing out from the ongoing background activity in the frequency range of interest (80–500 Hz) and having at least 10 ms inter-event interval. In order to distinguish real HFOs from the filtering effect of a sharp transient (130) or from other EEG activity, recent advances in the automatic detection algorithms have also incorporated information from (i) the time–frequency domain, assuming that an HFO must appear as a short-lived event with an isolated spectral peak at a distinct frequency (51, 122, 128, 130) (see Figure 4); and (ii) the unfiltered signal, assuming that a real HFO must be visible not only in the filtered but also in the raw signal (131).

### High-Frequency Oscillations and MEG

The non-invasive detection and localization of HFOs with MEG would significantly expand the clinical utility of these new promising biomarkers to an earlier stage in the diagnostic process and to a wider range of patients with epilepsy. Some recent studies have shown that epileptic HFOs can be detected non-invasively with scalp EEG (51, 110, 132–136), MEG (49–52, 137–142), or simultaneously with both techniques (51). Figure 4 shows simultaneous MEG and scalp EEG data with interictal HFOs co-occurring with epileptic spikes in a pediatric patient with focal epilepsy (51).

### Detection of Epileptic HFOs with MEG

Given the increasing interest in the non-invasive localization of HFOs using MEG, recent studies have proposed novel methods for the automatic or semi-automatic detection of HFOs and their distinction from artifacts in MEG recordings (49–51). MEG data contain more high-frequency artifacts than iEEG (143), thus a careful visual inspection of the data is required to guarantee that the detected HFOs are indeed of cerebral origin. A purely visual HFO detection has been used when a limited number of virtual sensors were investigated (49, 52) (more details about “virtual” sensors are provided in Section “Non-Invasive Localization of Epileptic HFOs with MEG”). Yet, given the lower rates of HFOs in the MEG data compared to the iEEG and the high number of physical sensors (>300), a purely visual approach is impractical when analyzing the MEG signals from all the physical sensors as in Ref. (50, 51). A semi-automated approach for the HFO detection is the most appropriate in these cases. This approach consists of a highly sensitive automatic detection followed by the visual review of the detected events by an EEG/MEG expert in order to increase the specificity of the detection method (28, 126, 127, 135).

The automatic detector used in Ref. (50) was designed to identify HFOs in all the MEG channels and to keep only the events occurring in the channel with the highest HFO rate. The proposed detector disregarded any HFO that (i) occurred simultaneously in more than 100 channels and in different frequency bands, since muscle activity and movement artifacts usually involve many channels and have a broad frequency content (144); (ii) occurred in channels located at the edge of the MEG helmet, since these channels are mostly affected by movement (143); or (iii) showed a power increase in the ripple band lower than in channels with no HFOs. These criteria were proposed to exclude any HFO occurring simultaneously with other high-frequency physiologic activity, instrumental noise, or artifacts, since they could affect the MSI results. Then, the visual review was performed to inspect the time–frequency map and to discard artifacts visible in the MEG signals in the clinical frequency range (0.3–70 Hz). Limitations of the proposed method are possibly due to the subjectivity of the time–frequency map inspection, as well as to the lack of consideration of peripheral recordings, such as electrocardiography (ECG), electromyography (EMG), or electrooculography (EOG), which can help the identification of cardiac, muscular, or ocular artifacts (145) resembling HFOs.

**Figure 5:**
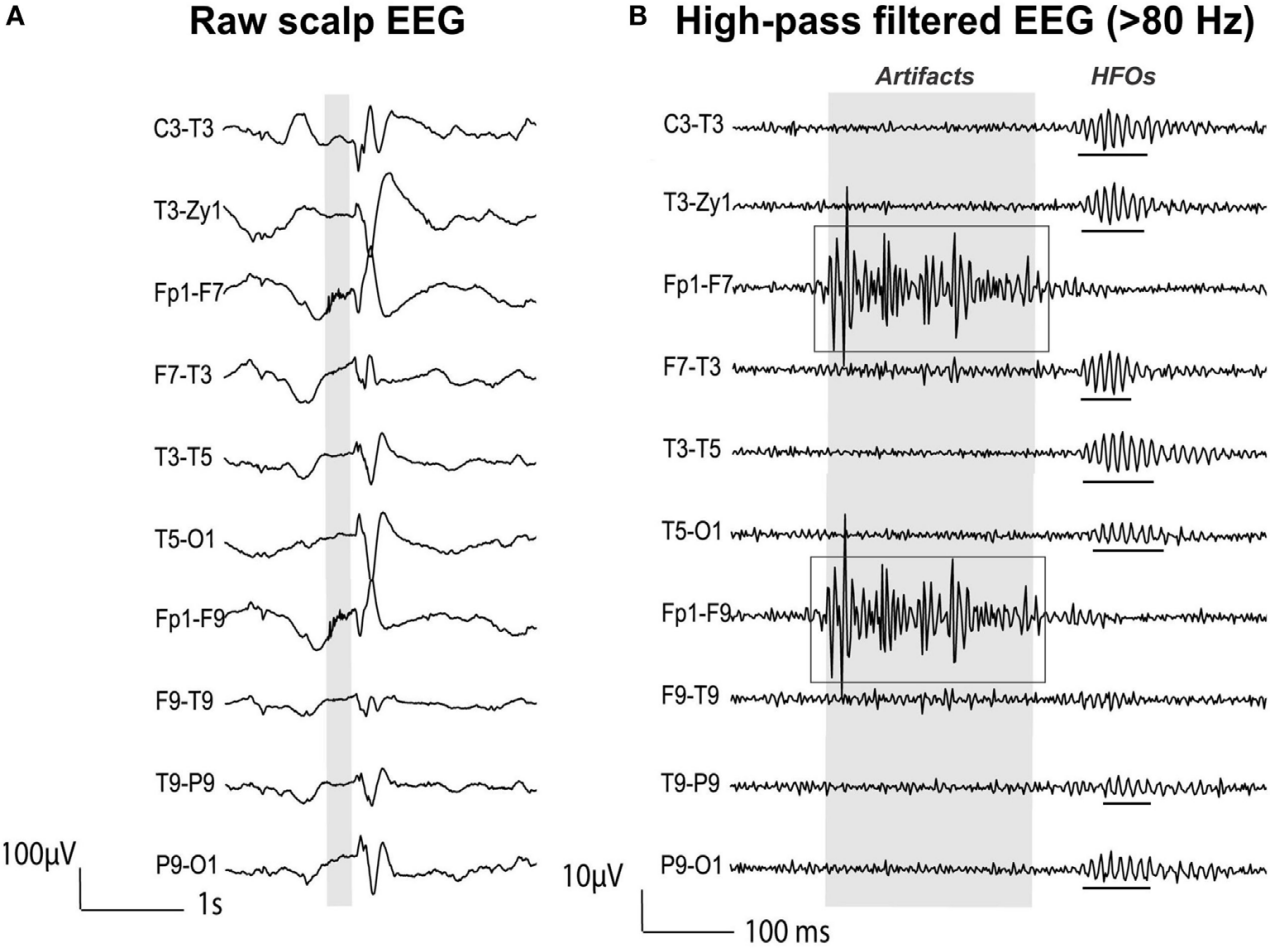
**High-frequency oscillations (HFOs) vs. artifactual oscillations recorded with scalp electroencephalography (EEG) data from a patient with focal epilepsy**. **(A)** Raw EEG with interictal epileptic spikes (gray section). **(B)** EEG filtered with high-pass filter of 80 Hz. Gray section in **(A)** is expanded in time and amplitude in **(B)**. Ripple HFOs are underlined. The morphology of HFOs is more rhythmic and regular in amplitude and frequency than artifactual oscillations. Source: adapted from Andrade-Valenca et al. (110).

Papadelis and colleagues (51) proposed a semi-automatic method to identify the HFOs that occurred on both MEG and scalp EEG data and that overlapped IEDs. The proposed detector identifies HFOs in the EEG signals not only looking at the time domain but also verifying the presence of an isolated island in the time–frequency map in order to automatically disregard possible artifacts (122, 130). Then, the visual review of the detected events was performed to keep only the HFOs that are also visible in the MEG data and that concur with the IEDs. In addition, to exclude any possible artifacts, the authors followed the guidelines proposed in Ref. (135) and excluded any oscillation: (i) with very high amplitude compared to the background; (ii) showing irregular morphology or large frequency variability (as shown in Figure 5); or (iii) co-occurring with muscle, movement, and electrode artifacts as identified with EOG, ECG, and EMG data at the timing of the detected HFOs. The main limitation of the proposed detection method is the lack of an automatic HFO detection in the MEG signals: the EEG/MEG expert should verify the presence of simultaneous HFOs in the EEG/MEG signals through visual inspection, which can be subjective and possibly biased.

Further studies on simultaneous EEG/MEG recordings (51), possibly accompanied by simultaneous iEEG as well (135), may allow a better understanding of the scalp HFOs. Further knowledge and agreement about the morphology and structure of the HFOs in MEG data may lead to the design of highly reliable detection algorithms. A fully automated approach for the HFO detection would enable to analyze large databases minimizing the selection bias and human labor.

### Non-Invasive Localization of Epileptic HFOs with MEG

Given the high resistivity of the skull and the assumption that a large extent of the cortex needs to be active in order to observe an HFO on the scalp, HFOs have been mostly investigated with intracranial depth electrodes and grids/strips on the cortical surface (26, 27, 40, 120). However, a few recent studies reported HFOs in the ripple frequency band recorded with scalp EEG in patients with epilepsy (51, 110, 132–135), showing lower HFO rates than in the iEEG. A recent scalp EEG study also reported fast ripples (250–500 Hz) recorded with subdermal electrodes in patients with focal epilepsy (136). Given the low number of electrodes used, source localization techniques have not been used to localize the HFO generators in all these scalp EEG studies, except for the one of Papadelis and colleagues (51) who localized the HFO sources from high-density scalp EEG data of children with MRE. High-density scalp electrode distributions indeed are necessary to obtain a solid sampling and accurate localization of HFOs on the scalp, as showed by Zelmann and colleagues (135), since such oscillations represent the sum of activity of multiple spatially distributed focal and coherent sources.

**Figure 6:**
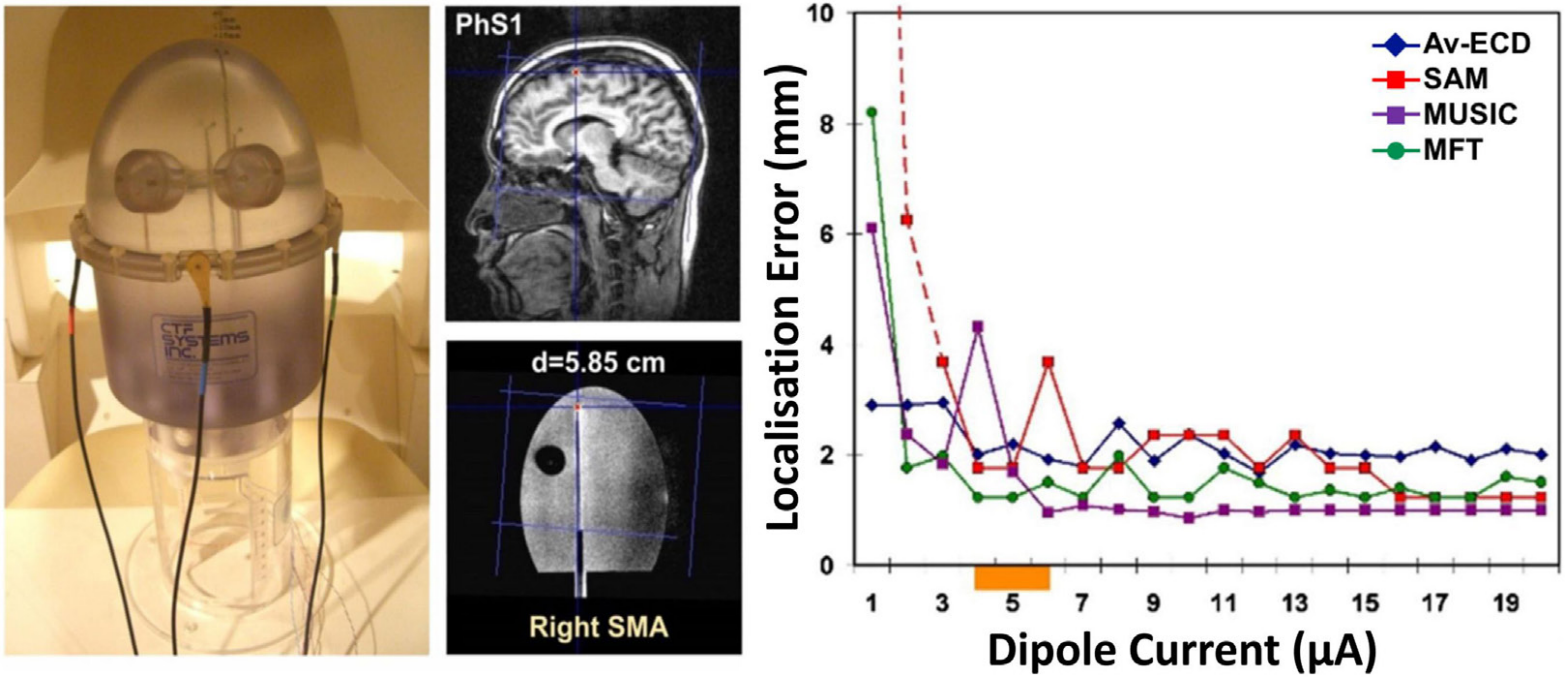
**Assessment of magnetoencephalography localization error for high-frequency oscillations (HFOs)**. Localization error (right panel) for a dipolar superficial source (central panel) implanted in a phantom construction (left panel) for different dipole currents (right panel, *x*-axis) and different source localization methods (left panel, different lines): equivalent current dipole on averages (Av-ECD, blue line), MUltiple SIgnal Classification (MUSIC, purple line), synthetic aperture magnetometry (SAM, red line), and magnetic field tomography (MFT, green line). The orange bar on the *x*-axis indicates the current corresponding to human HFOs. For details regarding the localization methods, the interested reader should refer to Ref. (48). Source: adapted from Papadelis et al. (48).

Over the last years, different research studies (48–52, 137–142) have focused on the possibility of recording HFOs with MEG and localizing their generators at the source level, given the significant inherent advantages of MEG compared to scalp EEG. The first evidence that HFOs can be non-invasively detected and localized using MEG with an accuracy of few millimeters was provided by Papadelis et al. (48). In this study, the authors used a head-shaped construction to generate artificial signals that resemble the human HFOs and used MSI with different source methods to localize the underlying generators (see Figure 6). Four different source localization methods were used: (i) the equivalent current dipole (ECD), which describes the underlying source as an infinitesimally small line current element (81), was applied at the peak activity of HFOs on averaged data; (ii) the MUltiple SIgnal Classification (MUSIC), which scans a single ECD through a three-dimensional head volume and computes projections onto an estimated signal subspace (146); (iii) the synthetic aperture magnetometry (SAM), which is a beamformer designed to detect signals from a specified location and attenuate signals from all other locations (147); and (iv) the magnetic field tomography (MFT), which relies on a non-linear algorithm with optimal properties for tomographic analysis of the MEG signal (148). The authors showed that weak transient signals, resembling the human HFOs, can be detected with an accuracy of few millimeters at cortical and subcortical regions. All localization methods showed high accuracy (localization error <5 mm) even when only few (six) trials were included in the analysis. In particular, the localization accuracy was unaffected by the low number of trials (six) for the beamformer (i.e., the SAM) and the MFT, while decreased for the MUSIC and ECD on averaged data. Although this study was the first one to provide evidence that human HFOs can be non-invasively localized with MEG, it did not report source localization data for single events. Epileptic HFOs are non-phase-locked spontaneous events and their localization analysis must be performed on single trials. Furthermore, the confounding effect of background activity unrelated to HFOs and its effect on the source localization accuracy were not considered.

The source localization of extended generators of oscillatory activity in specific frequency bands requires dedicated methodologies. Given the low amplitude and low SNR of HFOs, the source localization techniques commonly used for the analysis of epileptiform discharges with MEG (98, 149) (e.g., the ECD) are not recommended for the localization of HFOs (150, 151). This is because HFOs are not phase-locked events, and thus, their exact onset timing is unknown. As a result, different events cannot be averaged before solving the inverse problem to improve the SNR, as is typically done in stimulation studies (48, 152) or with epileptic spikes of similar morphology (41). Thus, alternative methods optimized for the source localization of single oscillatory events are necessary.

**Figure 7:**
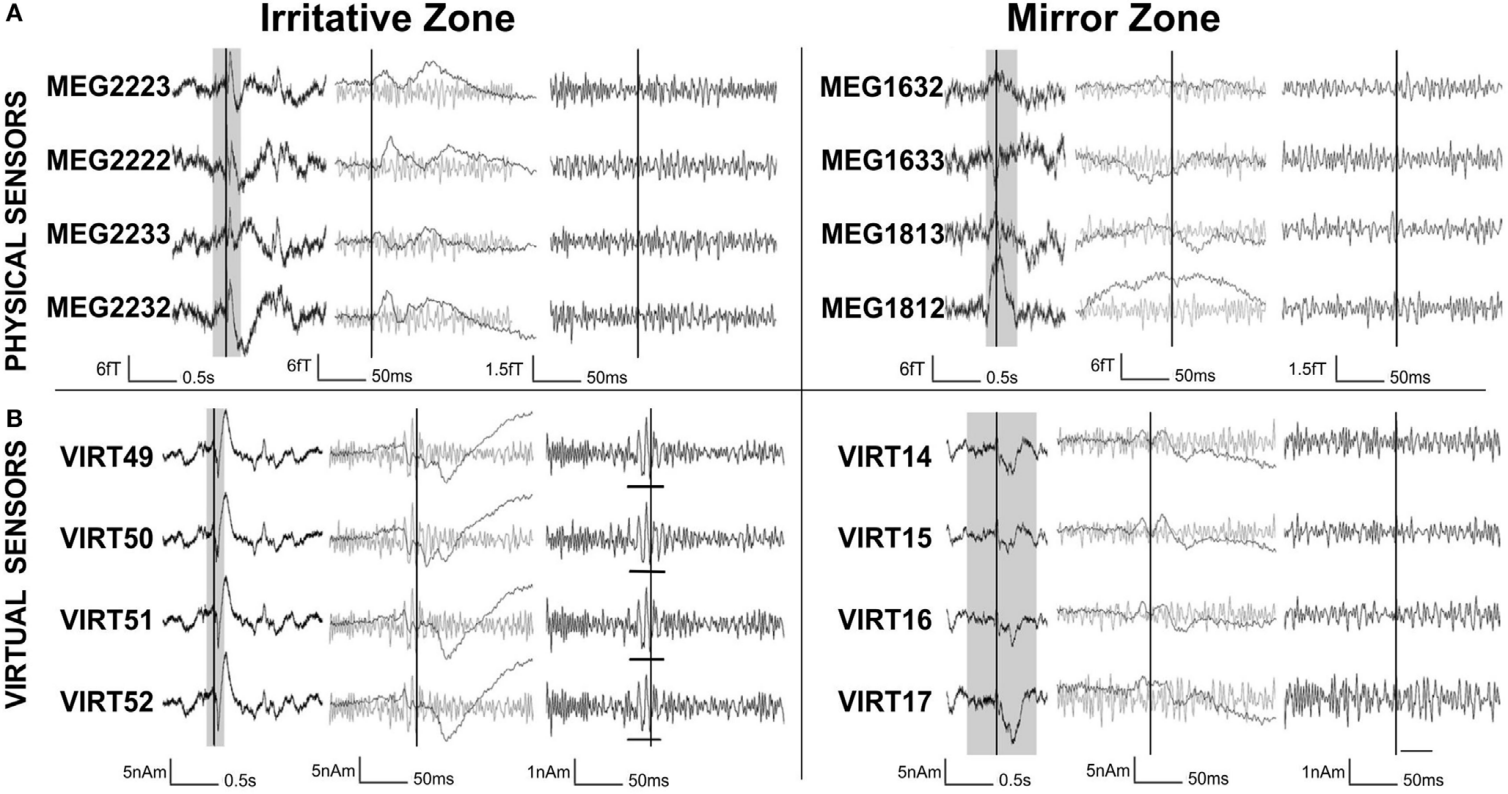
**High-frequency oscillations (HFOs) on physical magnetoencephalography (MEG) sensors vs. virtual sensors constructed using beamforming techniques**. Signals from physical MEG sensors **(A)** over the region where virtual beamformer sensors **(B)** were placed. Left: signals recorded from the irritative zone; right: signals from the mirror zone, i.e., homologous position in the contralateral hemisphere. The data around the interictal spikes (gray section) are stretched in time and high pass filtered (>80 Hz) to visualize HFOs. In the irritative zone, the virtual sensors [**(B)**, left] clearly show HFOs (underlined) that are not discernable in the physical sensors [**(A)**, left]. Neither spikes nor HFOs are visible in the physical and virtual sensors in the contralateral zone (right). Source: adapted from van Klink et al. (49).

To improve the SNR of HFOs, beamforming techniques have been used (49, 52). Beamformers have the potential to distinguish HFOs from noisy background activity (48, 137, 153). The beamformer method (154–156) reconstructs the neuronal activity for specific locations within the brain, so-called virtual sensors, as the weighed contribution from different MEG physical sensors. Such beamformer weights act like spatial filters and allow attenuating noise from distant sources (154–157). In a pioneer work, Van Klink et al. (49) used virtual sensors, whose location was defined based on the localization of the interictal spikes, in order to improve the SNR of the MEG signals in the time domain and to confirm the presence of HFOs (>80 Hz). The results of this study showed that the less noisy beamformer virtual sensors enabled visual detection of epileptic HFOs that could not be identified in the signals recorded by the physical MEG sensors (see Figure 7). The HFOs were detected more often in the irritative zone than in the contralateral “mirror” region and overlapped with other clinical findings. This study showed that the proposed non-invasive method enables visual identification of areas with epileptic HFOs using MEG. However, this study presented a methodological drawback: a limited number of virtual sensors were selected based on the location of spikes in the physical MEG sensors missing possible HFOs which may occur outside the area under investigation. The same approach was used by Nissen et al. (52) to show that brain areas with interictal spikes and HFOs were functionally isolated from the rest of the interictal epileptic network. The automatic placement of a larger number of virtual electrodes, along with automatic HFO detection, would make the proposed method more suitable for application in clinical setting (52).

**Figure 8:**
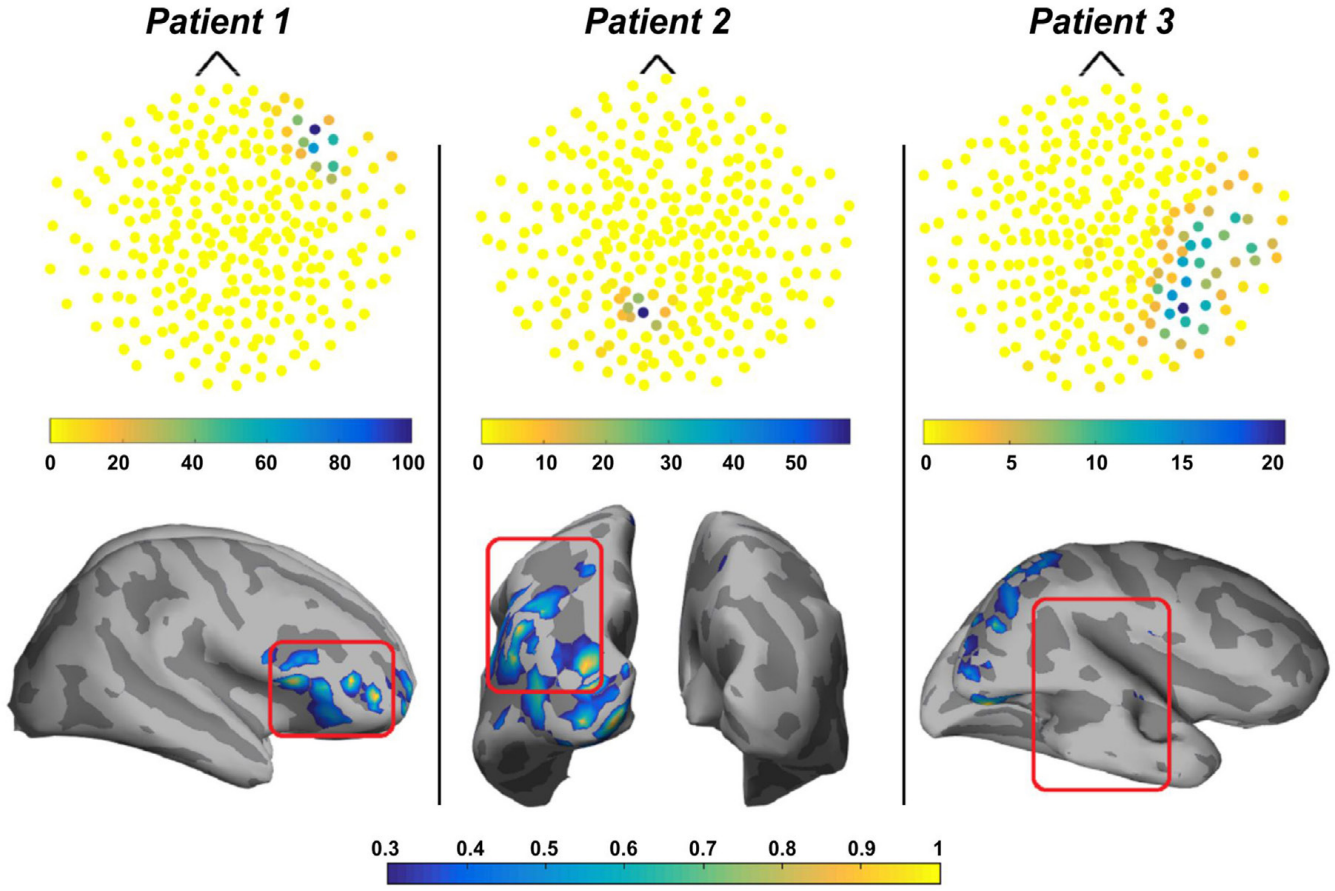
**Output of the detection and localization of high-frequency oscillations (HFOs) with magnetoencephalography (MEG) in three patients with focal epilepsy**. Upper panels: topographic maps of the HFO detection in the MEG sensors. Each sensor is represented by a dot whose color reflects the number of detected HFOs at the time of the HFOs in the highest rate channel. Lower panels: source localization (wavelet maximum entropy on the mean) of HFOs in the ripple band for the same three patients. Sources are displayed in a normalized color scale. The epileptogenic zone, determined based on the available clinical information, is delineated with a red square. For patient 1 and 2, the sources of HFOs were totally or partially concordant with the epileptogenic region, whereas for patient 3, they were discordant. Source: adapted from von Ellenrieder et al. (50).

Another practical approach for the source localization of ripple HFOs in MEG signals was proposed by von Ellenrieder et al. (50). This was the first study that detected interictal HFOs as visible events standing out from the background MEG signal in the time domain and localized their generators at the source level. The authors used the wavelet maximum entropy on the mean (wMEM) method for the source localization. The wMEM is an extension of the maximum entropy on the mean (MEM) method (158–160) that is particularly well suited for this application since it was developed for the localization of oscillatory activity (161). The wMEM decomposes the signal into a discrete set of wavelet bases functions; it then performs MEM source localization on each time–frequency box (161). The study of von Ellenrieder and colleagues (50) is the first one performing MSI of spontaneous, and likely pathological, HFOs the ripple band (80–160 Hz) in patients with epilepsy. They detected HFOs from the signals of the MEG physical sensors, independently of the interictal spikes, and used the wMEM method to determine the location and extent of their generators on the cortex. For the validation of the HFO localization, the authors evaluated the spatial concordance of the HFO sources with the epileptogenic region defined by two specialists based on the available clinical information for each patient (i.e., resected region, ictal and interictal iEEG findings, visible lesion in the MRI, ictal, and interictal scalp EEG findings) (see Figure 8). The validation did not show fully concordant results suggesting the need for further studies in a larger cohort of patients. Furthermore, such a validation approach does not prove that non-invasively localized HFOs provide the same results as invasively localized HFOs. To this purpose, comparisons of MEG results with the iEEG findings from the same subject are necessary to verify that MEG HFO localization coincides with the location obtained with invasive methods.

**Figure 9:**
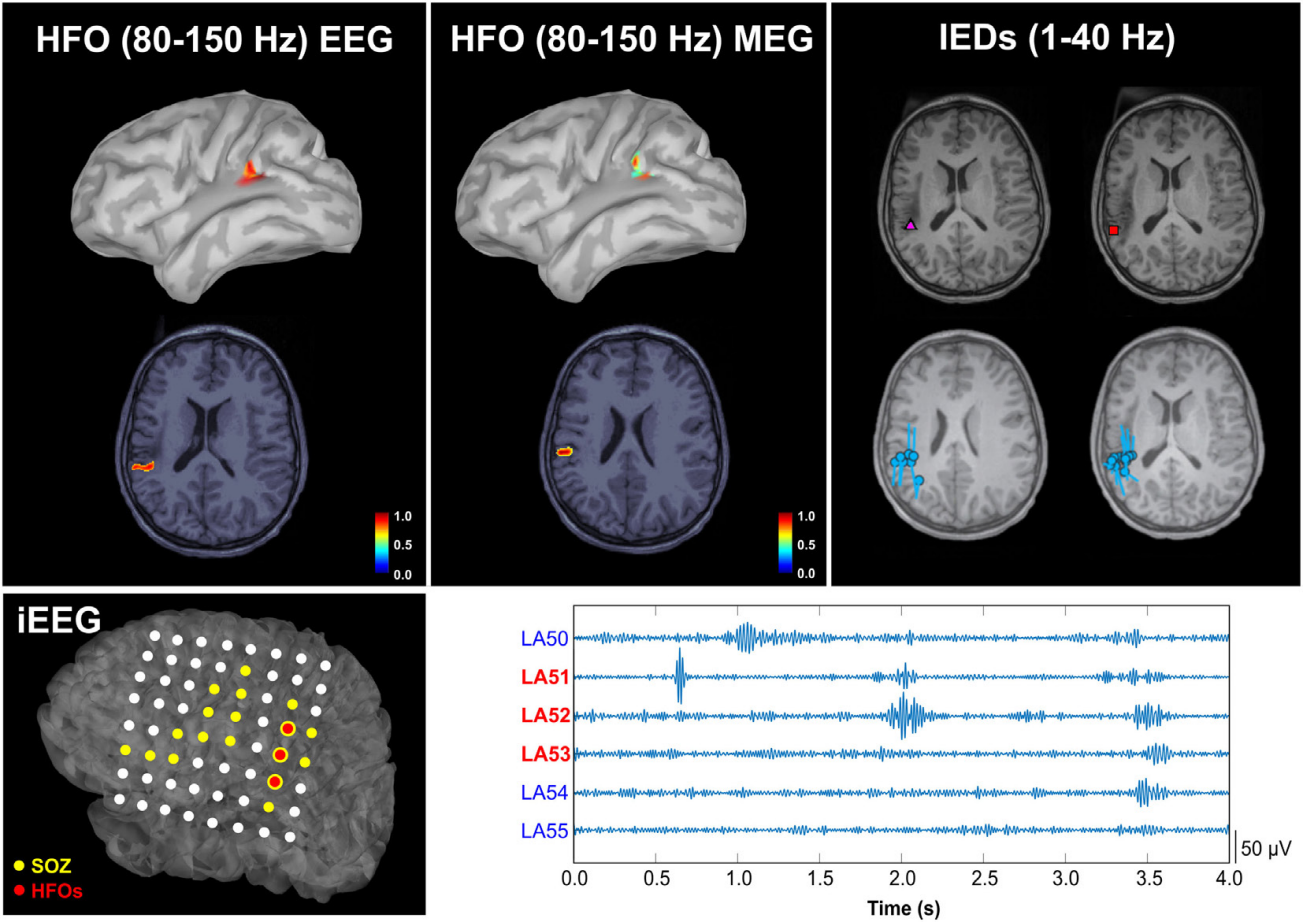
**Spatial concordance between the high-frequency oscillation (HFO) zone localized non-invasively with magnetoencephalography (MEG) and high-density scalp electroencephalography (EEG), the irritative zone, and the HFO zone defined invasively with intracranial EEG (iEEG)**. Upper panel: localization of HFOs with scalp EEG (left) and MEG (middle), and localization of interictal epileptiform discharges (IEDs) with MEG (right) overlaid on patient’s magnetic resonance imaging (MRI). The HFO source map represents the normalized activation values thresholded at 60% of the maximum activity. Cyan circles and bars indicate the locations and orientations of the equivalent current dipoles. The purple triangle indicates the location of averaged dipoles, and the red rectangle the location of the dipoles from averaged IEDs. Lower panel: on the left, the localization of HFOs on the iEEG grid is reported (three electrodes with the highest HFO rate are highlighted in red on the map of the grid implanted on the patient’s left cortex); on the right, 2 s of data from these three channels with the highest HFO rate are shown (LA51, LA52, and LA53). The cortical location corresponding to the electrodes was determined by co-registering the post-implanted computed tomography and MRI. Source: from Papadelis et al. (51).

The same method for the localization of ripple HFOs was also used by Papadelis and colleagues (51) who were the first to investigate interictal HFOs with simultaneous MEG and high-density scalp EEG data from pediatric patients with MRE. In this study, the authors identified the HFOs occurring with interictal spikes in both MEG and scalp EEG and performed source localization using wMEM on the data from both modalities. The results of this study showed that the HFO localization was concordant between MEG and scalp EEG as well as concordant with other clinically relevant zones, such as the irritative zone and the SOZ (see Figure 9). The HFO localization for one patient was further validated against the ground truth given by the findings obtained from the HFOs detected in the iEEG data (see Figure 9, bottom). Such validation confirmed the source localization reliability of both MEG and scalp EEG. However, given the limited sample size, these promising findings need further validation.

In addition to the studies described so far, a series of MEG studies investigated the power in high-frequency bands of patients with epilepsy using accumulated source imaging (137–142). The accumulated source imaging is a source localization method that was developed to localize and quantify spontaneous brain activity (162). Researchers showed that MEG high-frequency components were localized within the EZ in pediatric patients with epilepsy (137–142). Xiang and co-workers (139, 140) also reported that MEG source localization of ictal and interictal high-frequency components was concordant with the iEEG results. The main limitation of these studies (137–142) is that they did not investigate HFOs as individual events in the time domain, as typically done with iEEG. Thus, it is not possible to establish whether the reported high-frequency components have the typical HFO morphology in the time domain and if they represent the same phenomenon observed in the time domain signals (27, 120, 135).

In summary, evidences from phantom and human studies (48–52, 137–141, 143) have shown that human HFOs can be non-invasively localized using MEG with an accuracy of few mm. Proper methods should be used for their localization because HFOs are weak oscillatory transients, which can be otherwise undesirably removed if localization methods are applied on averaged or coregistered events, as HFOs are not necessarily mutually phase locked. To date, there is no MEG study that has evaluated the clinical value of the HFO source localization in patients with MRE. To this purpose, further studies are needed to correlate the localization of the HFO zone with the resected area and the postsurgical outcome of patients with MRE. In addition, systematic investigations of simultaneous MEG, high-density scalp EEG, and iEEG would allow a direct comparison of the two non-invasive modalities with the invasive gold standard for an optimal validation of the HFO source localization results.

## Discussion

Significant advances in diagnostic technology have largely improved efficacy and safety of epilepsy surgery. Yet, there is still an overriding need to identify and validate new reliable biomarkers that can precisely delineate the extent and location of the EZ. The identification of a more specific biomarker of epileptogenicity would (i) limit the need for long-term monitoring, (ii) enable a smaller and more precise resection of the EZ, (iii) improve the postsurgical outcome, and (iv) reduce the neurological deficits due to excessive surgical resection volume (37). To this purpose, HFOs have been the focus of investigations in the last decade given their emerging potential as a new precise and reliable biomarker of epileptogenicity (26, 27). The possibility to record such biomarkers with non-invasive techniques is crucial to expand their clinical utility (40). The non-invasiveness of the acquisition technique would (i) limit the need for presurgical long-term monitoring with iEEG; (ii) enable an optimized planning of the intracranial investigations; (iii) allow the assessment of the efficacy of therapeutic interventions without waiting for a seizure to occur, which can be associated with significant morbidity or mortality; (iv) permit definitive differential diagnosis of epilepsy from acute symptomatic seizures so treatment can begin immediately; and (v) enable the study of the differentiation between physiologic and pathologic HFOs by allowing the non-invasive investigation of healthy controls. This strong interest in localizing HFOs combined with MEG’s increasing role in the presurgical evaluation of patients with MRE (41–47) has led to increasing interest in detecting and localizing epileptic HFOs using MEG (48–52, 137–142).

In this review, we provided an overview of recent MEG studies that investigated HFOs in patients with epilepsy, discussing the proposed methodologies and the main findings reported in the current literature. The reported results are promising, but also foster the need for further MEG studies aiming at: (i) evaluating the accuracy of the HFO source localization against a ground truth given by simultaneous iEEG recordings in a large sample of patients, (ii) evaluating the clinical value of MEG in the HFO localization by looking at the patient’s postsurgical outcome and its correlation with the resection of the HFO generating areas; and (iii) establishing a definition of HFOs that is appropriate for automatic detection in the context of MEG studies (as this could require different criteria compared to the iEEG literature), and possibly validating it against the patient’s outcome, rather than visual detection (118). Moreover, since high SNR is critical for the accurate localization of HFOs (48), the use of virtual channels (46, 49) should be further investigated. This will allow the localization of HFOs without relying on the spike location and avoiding biased results. Source localization could also be improved by merging simultaneous scalp EEG and MEG recordings: the complementary information of these two modalities might improve the source localization reliability (163), in particular for the MEM method (164).

## Conclusion

The emerging potential of MEG for the detection and localization of HFOs in patients with epilepsy has been reported in several recent studies. The promising findings emphasize the need for large and robust studies necessary to establish and widespread the clinical use of HFOs in MEG data as a non-invasive biomarker of epileptogenic tissue. The possibility to detect and localize HFOs with MEG would open new possibilities of diagnosing and monitoring epilepsy. Non-invasively recorded HFOs could provide clinicians with an early epileptogenic marker in all patients with epilepsy, as well as in patients at high-risk of epilepsy, and could be also used to evaluate the effect of antiepileptic drug therapy. Finally, further studies are needed to address the question of whether the resection of the HFO zone localized with MEG can predict the postsurgical outcome of patients with MRE.

## Author Contributions

ET designed and drafted the work. She approved the version to be published and agreed to be accountable for all aspects of the work. JM contributed to the conception of the work and revised it critically for important intellectual content. He approved the version to be published and agreed to be accountable for all aspects of the work. PG contributed to the conception of the work and revised it critically for important intellectual content. She approved the version to be published and agreed to be accountable for all aspects of the work. PP contributed to the conception of the work and revised it critically for important intellectual content. He approved the version to be published and agreed to be accountable for all aspects of the work. CP contributed significantly to the conception of the work and revised it critically for important intellectual content. He approved the version to be published and agreed to be accountable for all aspects of the work.

## Conflict of Interest Statement

The authors declare that the research was conducted in the absence of any commercial or financial relationships that could be construed as a potential conflict of interest.

## Abbreviations

ECG, electrocardiography; EEG, electroencephalography; EMG, electromyography; EOG, electrooculography; EZ, epileptogenic zone; fMRI, functional magnetic resonance imaging; HFO, high-frequency oscillation; IED, interictal epileptiform discharge; iEEG, intracranial electroencephalography; MEG, magnetoencephalography; MEM, maximum entropy on the mean; MRE, medically refractory epilepsy; MRI, magnetic resonance imaging; MSI, magnetic source imaging; MUSIC, MUltiple SIgnal Classification; PET, positron emission tomography; SAM, synthetic aperture magnetometry; SNR, signal-to-noise ratio; SOZ, seizure-onset zone; SPECT, single photon emission computed tomography; wMEM, wavelet maximum entropy on the mean.
